# Ethnobotanical survey of medicinal plants used as insects repellents in six malaria endemic localities of Cameroon

**DOI:** 10.1186/s13002-017-0155-x

**Published:** 2017-06-08

**Authors:** Roger Ducos Fokouo Youmsi, Patrick Valère Tsouh Fokou, Elisabeth Zeuko’o Menkem, Issakou Bakarnga-Via, Rodrigue Keumoe, Victor Nana, Fabrice Fekam Boyom

**Affiliations:** 1 Laboratory for Phytobiochemistry and Medicinal Plants Studies, Department of Biochemistry, Faculty of Science, University of Yaoundé I, P.O. Box 812, Yaoundé, Cameroon; 2 Department of Biology, Faculty of Science, University of Adam Barka-Abeche, P.O. Box 1173, Abeche, Chad; 3 National Herbarium of Cameroon, P.O. Box 1601, Yaoundé, Cameroon

**Keywords:** Ethnobotanical knowledge, Malaria, Insects, Repellent plants, Mosquitoes

## Abstract

**Background:**

The combined efforts to combat outdoor/indoor transmission of malaria parasites are hampered by the emerging vector resistance in a wide variety of malaria-endemic settings of Africa and the rest of the world, stressing the need for alternative control measures. This study aimed at documenting insect’s repellent plant species used by indigenous populations of 6 localities of East, South, West and Centre regions of Cameroon.

**Methods:**

Information was gathered through face-to-face interviews guided by a semi-structured questionnaire on the knowledge of medicinal plants with insect repellent properties.

**Results:**

A total of 182 informants aged from 25 to 75 years were recruited by convenience from May to June 2015. The informants had general knowledge about insects’ repellent plants (78.6%). A total of 16 plant species were recorded as insects’ repellents with 50% being trees. The most cited plants were *Canarium schweinfurthii* (Burseraceae) (in four localities, 58/182), *Elaeis guineensis* (Arecaceae) (in three localities, 38/182), *Chromolaena odorata* (Compositae) (16/182) and *Citrus limon* (Rutaceae) (11/182) in two localities each. Among the repellent plant species recorded, 50% were reported to be burnt to produce in-house smokes, 31.2% were mashed and applied on the body, and 18.8% were hung in the houses. The leaf was the most commonly used plant part (52.9%), followed by the bark (17.6%).

**Conclusions:**

This study has shown that rural populations of the 6 targeted localities possess indigenous knowledge on repellent plants that are otherwise cost-effective and better choice for repelling insects including malaria-transmitting mosquitoes. Meanwhile, such practices should be validated experimentally and promoted as sustainable malaria transmission control tools in the remotely located communities.

**Electronic supplementary material:**

The online version of this article (doi:10.1186/s13002-017-0155-x) contains supplementary material, which is available to authorized users.

## Background

Malaria is among the major vector-borne diseases that exact the heaviest toll from populations of sub-Saharan Africa. Worldwide, it is estimated that about 3.4 billion people are at risk of malaria. Within the past few years, approximately 207 million yearly cases of malaria occurred globally with the highest burden (80% of cases and 90% of deaths) recorded in Sub-Saharan Africa. The huge proportion of deaths (77%) occurs in children under 5 years [1]. The whole Cameroonian population is at risk of malaria transmission, of which 71% are at high risk and 29% at low risk. *Anopheles gambiae* is the main vector responsible for malaria transmission in the sub-Saharan Africa region [2].

The combined efforts of distributing long lasting insecticide treated mosquito nets, indoor residual sprays, and effective case management with potent antimalarials have dramatically declined the malaria related illness and mortality [3]. However, the phenomenon of insecticide resistance poses a serious threat to sustainable insecticide-based vector control in many African countries. Besides, the lack of effective anti-vector tools designed specifically to prevent outdoor transmission and the serious threat posed by insecticides to the environment as well as the human health emphasize the need to develop new vector control approaches that can complement the existing interventions. In that framework, there are increased research efforts to develop natural and environment friendly interventions including plant-based mosquito repellents. Of note, plant-based repellents are generally target-specific and relatively non-toxic, and are still extensively used traditionally as first intention and affordable tools in malaria endemic rural communities for protection against mosquito bites [4].

Therefore, the indigenous knowledge and use of plants as repellents should be documented and preserved as a cheap and sustainable way of preventing mosquito's bites or entering homes in Cameroon and beyond. This documentation represents a prerequisite for future research on efficacy and safety as well as identification of single chemical entities with mosquito repellent activity which could lead to the development of standardized bio-pesticides. So far, only a limited number of studies have been conducted in Cameroon on traditional use of medicinal plants to repel insects/mosquitoes [5]. The question is therefore to know if there is a consensus extent in indigenous use of plants from the Cameroonian biodiversity to repel mosquitoes/insects. To fill this gap in, we hypothesized that plants from six selected localities of the East, South, West and Centre regions of Cameroon are culturally used to repel mosquitoes/insects. Thus, the present paper documents the indigenous knowledge on plants used as mosquito/insect repellents in the six selected localities in order to further evaluate their potential for new plant-based repellent products.

## Methods

### Selection of the study sites

**Fig. 1:**
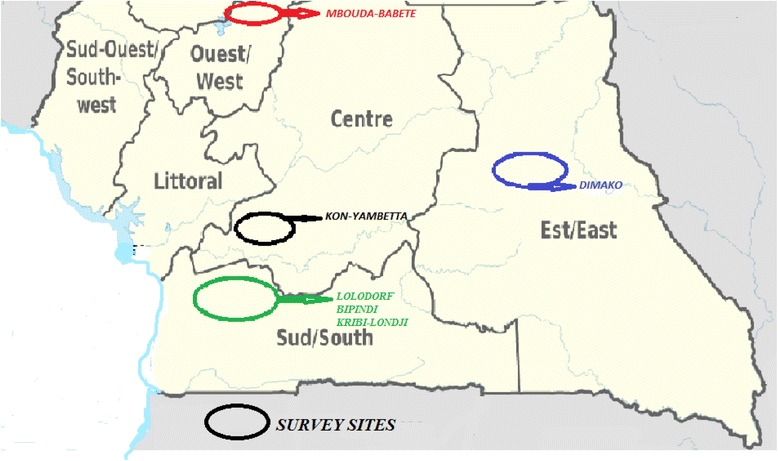
Study sites of the ethnobotanical survey of insect repellent medicinal plants. Study sites were selected based on their ethno-cultural and floristic characteristics and included Dimako (East region), Kon-Yambetta (Centre region), Lolodorf, Bipindi, and Kribi-Londji (South region), and Mbouda-Babete (West region)

The study sites were selected among the malaria endemic regions of Cameroon. In fact, the 71% Cameroonian population that are at high risk of malaria transmission [2] live in the endemic and perennial zones of continuous transmission (7 to 12 months) that cover the Southern Cameroonian Equatorial forest, the High Western Plateau altitude and the Coastal region, including all the selected study sites where about a hundred infective bites per man per month can be registered [6]. Besides, the presence of indigenous pygmies [Lolodorf, Bipindi (South region), Dimako (East region)] who have great knowledge of the forest, plants and their medicinal properties [7, 8], and the presence of herbal practitioners/medicinal plant users in Londji (South region), Mbouda-Babete (West region), and Kon-Yambetta (Centre region) also motivated their selection as study sites (Fig. 1).

### Lolodorf, Bipindi, and Kribi-Londji

Lolodorf, Bipindi, and Kribi-Londji are districts of the Ocean Division in the South Region of Cameroon nearby the western coast of Africa. The mean temperature varies from 25 to 26 °C with two rainy and two dry seasons. Lolodorf is located between Ngoumou and Bipindi, in a zone of the Atlantic Littoral Evergreen Forest, extending over the equatorial climatic zone. Londji is a small locality located 20 km to North of Kribi District also characterized by a mangrove forest ecosystem, and has vast natural beaches and the largest fishermen village and fish market in Cameroon [9]. The Londji bay is an extension of the Atlantic Ocean on the continent and the mouth of the Lokoundje and Nyong rivers. The village showcases a vast diversity of heterogeneous but integrated populations and ethnic groups, as the dynamism of the fishing activity has attracted people from West Africa throughout the years. Londji prides itself in the sustainable preservation of the mangrove areas and wetlands. Moreover, it is crossed by dotted rivers with mangrove plots that promote the development of insects. The humidity remains high throughout the year and causes increased insects breeding sites [10]. Bipindi and Lolodorf are notable for being the home of Pygmy clans and their camp settlements and hunting areas, such as those of Lala, Bakola and Bagyeli pygmies. There are also settled Bantu people such as Ngumba and Kwassio tribes. Farming, tapping palm wine, gathering fruits and nuts for food, fishing, and hunting are the main occupations within these communities. Malaria and sleeping sickness both vector-borne diseases are common problems in this humid climate. Bagyeli and Bakola pygmies have great knowledge of using plants for their medicinal properties [7, 8]. The present study was partly conducted in Ngoyang, Mbikiliki, Mougoué, Ngovayang, and Bigambo, all surrounding villages of Lolodorf as well as in Bipindi and Bidjouka, two villages of Bipindi District.

### Dimako and surroundings

Dimako is a small town situated in the Upper Nyong Division of the East Region of Cameroon. It lays a little way south of East Region capital of Bertoua at only about 28 km away. Vegetation is characteristic of a tropical rain forest, dotted with fallows, which covers 90% of the municipal territory. The remaining 10% is covered by a shrubby savannah in the north of Dimako. A greater part of the forest is described as semi-caducus forest made of Sterculiaceae and Ulmaceae plants. Inhabitants of the Dimako District are very closely linked to the forest and its resources. Dimako District accounts 30 villages including Loussou and Mayos inhabited by Baka Pygmies. Due to the humid mosquito- and black fly-infested forests [11], the area was also selected as study site.

### Kon-Yambetta

Kon-Yambetta is a District of the Mbam-et-Inoubou Division (Centre region) located about 150 km (Northwest) from Yaoundé, in the grasslands between Bafia and Ndikinimeki. It is the transition zone between the forest and the savannah. The area has an equatorial type climate with two raining and two dry seasons. The inhabitants practice peasant agriculture dominated by the cocoa and diverse subsistence crops. Environmental degradation and mismanagement has increased the development of vectors (mosquitoes, mango flies) of diseases such as malaria. This area has Guinea savannah-type flora where the yambetta and bamoun people rely on herbs for first intention treatment when ill [12].

### Mbouda (Babete)

Babete is one of the 6 villages of Mbouda District (Bamboutos Division, West Region) located at approximately 3.2 km from Mbouda. It is characterized by high lands, cool temperatures, heavy rainfall, and savanna vegetation. The climate is classified as a tropical savanna, with a subtropical moist forest biozone [13]. This area is known for use of medicinal plants as source of essential oils as well as dry fruits and natural drinks. Such products are commonly manufactured and sold by the nuns of the Saint Benoît Monastery [13].

### Interview and collection of plants specimens

In prelude to the survey, legal authorities (village heads) from each study site were approached and the aims and objectives of the survey were discussed for authorization to investigate within their communities. Volunteers and recommended herbal practitioners were identified as potential informants and subsequently participated in personal interviews.

During a face-to-face interaction, the purpose and the procedure of the study, as well as the expected benefits and rights to their community were explained to them. The interviews were conducted in French or in the local languages with the assistance of volunteer interpreters when needed. The information surveys took place between May and June, 2015. A total of 182 informants from 38 households including local herbal practitioners were surveyed with a set of pre-tested semi-structured questionnaires in all the six study areas and selected for further interaction. Prior informed consent was obtained verbally from informants before they were interviewed.

During the interviews, semi-structured questionnaires were used to obtain information on plants used to repel mosquitoes/insects, including parts used, local name, modes of preparation and administration (Additional file 1). Some repellent plants were also identified by visiting the field with the informant (field interview); listening to him and asking questions; presenting plant specimens to informants and asking questions (plant interview); or using a checklist (checklist interview; plant name-common or vernacular-was addressed to the informants in order to gather supplementary field information). Group interview have sometimes developed spontaneously with community members and this helped to supplement the interview. Specimens were collected from the plants claimed by informants to have repellent activity. Such plants were identified by a taxonomist and voucher specimens deposited under specific identification numbers at the Cameroon National Herbarium in Yaoundé.

### Data analysis

For each cited plant species, the frequency of citation (FC) per locality was determined as:

$$ \mathrm{F}\mathrm{C} = \left(\mathrm{Number}\ \mathrm{of}\ \mathrm{citations}\ /\mathrm{Total}\ \mathrm{number}\ \mathrm{of}\ \mathrm{citations}\ \mathrm{for}\ \mathrm{all}\ \mathrm{recorded}\ \mathrm{species}\right)\ \mathrm{x}\ 100 $$

The value of FC obtained directly correlates with the broad use of the plant species [14].

## Results

### Profile of the informants

A total of 182 informants from the six localities agreed to participate in this study. Their profile is given in Table 1.

**Table 1:** Profile of informants in the study localities

Locality	Number of informants	Sex		Age range (years)	Informants with knowledge about repellent plants
		Men	Women		
Lolodorf and surroundings	53	48	5	25-70	51
Bipindi and surroundings	35	30	5	25-55	25
Londji	13	13	0	60-65	0
Kon-yambetta	32	29	3	25-75	18
Dimako and surroundings	36	36	0	25-55	36
Babete	13	2	11	40-60	13
Total	182	158 (86.8%)	24 (13.2%)		143 (78.6%)

Informants were recruited following a pre-tested semi-structured questionnaire in all the six study areas. Prior informed consent was obtained verbally from informants before they were interviewed

The age of respondents averaged 50 years, 86.8% being men and 13.2% women. Globally, 78.6% of respondents had knowledge about mosquito and/or insect repellent plants. The level of this knowledge varied from one locality to another with 100% in Dimako and Babete, 96.2% in Lolodorf and surroundings, 71.4% in Bipindi and surroundings, and 56.3% in Kon-yambetta. No specific record about mosquito and/or insect repellent plants was obtained from the Kribi-Londji site.

### Diversity of medicinal plants and knowledge on mosquito repellent plants

A total of 16 plant species belonging to 15 genera and 14 families were reported to be commonly used as insect repellents in the targeted communities as shown in Table 2. They consisted of 50% of trees, 31.2% of shrubs, and 18.8% of herbs. Of these, 56.2% were wild while 43.8% were usually cultivated. Lamiaceae, Leguminosae, Musaceae and Poaceae (2 species each) were the most represented families, while the others consisted of a single species each.

**Table 2:** Plants commonly used to repel insects in six Cameroonian local communities

Plant family	Species	Specimen number	Vernacular name	Origin	Botany	Part used	Mode of Preparation	Mode of administration
Annonaceae	*Greenwayodendron suaveolens* Engl. & Diels	1879 HNC	Mpolè (Bakola, Bagyeli)	Wild	Tree	Bark	Smash fresh bark	Skin application
Arecaceae	*Elaeis guineensis* Jacq.	34163 HNC	Lélendé (Bakola); Mimbang malendé (Bagyeli); Alen (Ewondo); Zam-na-malendé (Ngumba)	Cultivated	Tree	Flower	1. Burn dry flowers 2. Burn dry flowers + fresh leaves of *C. odorata.* 3. Burn dry flowers + leaves of *S. officinarum* + suspended termitarium	Smokes
Burseraceae	*Canarium schweinfurtii* Engl.	16929 SFR/CAM	Bélè (Ngumba); Ottou (Ewondo); Bologa (Baka); Beul (Bakoum); Waate (Yambeta)	Wild	Tree	Sap	Burn dry or fresh sap/mix ash with body milk/lotion.	Smokes/skin application of resulting potion
Compositae	*Chromolaena odorata* (L.) R.M.King & H.Rob.	57408 HNC	Gomgom (Ngumba)	Wild	Herb	Leaf and whole plant	1. Burn whole plant 2. Burn fresh leaves + dry flowers of *E. guineensis.*	Smokes
Lamiaceae	*Ocimum gratissimum* L.	5817SFR/Cam	Massissip (Ngumba)	Cultivated	Herb	Leaf	Smash leaves	Skin application
	*Premna angolensis* Gürke		Kinoubeunoubeu (Bafia)	Wild	Shrub	Leaf	Hang fresh leaves	Hang inside the house
Leguminosae	*Cylicodiscus gabunensis* Harms	21574 SRF/Cam	Okan; Adoum (Ewondo)	Wild	Tree	Bark	Burn dry bark + dry flowers of *E. guineensis*	Smokes
	*Erythrophleum ivorense* A.Chev.	45333 HNC	Mbamda (Bafia)	Wild	Tree	Bark	Burn fresh bark	Smokes
Musaceae	*Musa* ^*x*^ *paradisiaca* L.	42391 HNC	Ekouan (Ewondo)	Cultivated	Tree	Leaf	Burn dry leaves + flowers of *E. guineensis*	Smokes
	*Musa sp.*		Odué (Ewondo)	Cultivated	Tree	Leaf	Burn dry leaves + flowers of *E. guineensis*	Smokes
Piperaceae	*Piper umbellatum* L.	42274 HNC	Ndembèlembè (Baka)	Wild	Shrub	Leaf	Smashed fresh leaves	Skin application
Poaceae	*Saccharum officinarum* L.	25820 SRF/Cam	Nkogo’o (Bakola)	Cultivated	Shrub	Leaf	Burn fresh leaves + dry flowers of *E. guineensis +* suspended termitarium	Smokes
	*Cymbopogon nardus* (L.) Rendle		Citronella grass (English)	Cultivated	Herb	Whole	1. Hang whole plant 2. Distillate whole plant for essential oil	Hang/spray oil inside the house
Rutaceae	*Citrus limon* (L.) Osbeck		Nyopiang (Ngumba)	Cultivated	Tree	Fruit	Pressed fruit for juice.	Skin application
Siparunaceae	*Glossocalyx longicuspis* Benth.	46245 HNC	Anyanzoa (Ngumba)	Wild	Shrub	Leaf	Hang/Crumple fresh leaves	Hang/spread inside the house.
Zingiberaceae	*Aframomum alboviolaceum* (Ridl.) K. Schum.	34888 HNC	Ndjii (Baka)	Wild	Shrub	Leaf	Smash fresh leaves	Apply on skin

Information on medicinal plants uses was collected through visiting the field with informants, presenting plants specimens, and/or checklist interviews on medicinal plants used to repel insects, including mosquitoes. The plant parts used as well as the modes of preparation and administration were recorded

Overall, the majority of plant species were recorded in Lolodorf and Bipindi. The most cited were *Canarium schweinfurthii* recorded in four localities (Lolodorf, Bipindi, Kon-Yambetta, Dimako), and mentioned by 58 informants, followed by *Elaeis guineensis* cited in three localities (Lolodorf, Bipindi, Kon-Yambetta) and mentioned by 38 informants, *Citrus limon* and *Chromolaena odorata* cited in two localities (Lolodorf, Bipindi) and mentioned by 11 and 16 informants respectively. *Afromomum alboviolaceum* (Dimako), *Cymbopogon nardus* (Babete), *Cylicodiscus gabunensis* (Lolodorf), *Erythrophleum ivorense* (Dimako), *Glossocalyx longicuspis* (Lolodorf), *Musa paradisiaca* (Lolodorf), *Musa sp.* (Lolodorf), *Ocimum gratissimum* (Bipindi), *Piper umbellatum* (Dimako), *Premna angolensis* (Kon-Yambetta), and *Saccharum officinarum* (Lolodorf) were recorded only in one locality, but no clear patterns could be identified to explain this variation (Table 3). This inter-community variation in knowledge and use of insect repellent medicinal plants could result from the fact that ethnobotanical knowledge is not shared among communities. This important gap could also be due to a lack of individual motivation, experience, or curiosity of inhabitants to search beyond the customary habits of the surveyed communities [15]. The ethnobotanical knowledge appeared to be commonly shared among people from the same setting rather than between communities.

**Table 3:** Frequency of Citation (FC in %) of plant species in the survey areas

Percent Frequency of Citation (in %)							
Plant family	Species	Lolodorf	Bipindi	Kon-Yambetta	Dimako	Londji	Babete
Annonaceae	*Greenwayodendron suaveolens*	0	12.6	0	0	0	0
Arecaceae	*Elaeis guineensis*	25.2	14.6	32.5	0	0	0
Burseraceae	*Canarium schweinfurthii*	32.2	36.9	49.8	34.2	0	0
Compositae	*Chromolaena odorata*	8.8	17.9	0	0	0	0
Leguminosae	*Cylicodiscus gabunensis*	2.9	0	0	0	0	0
	*Erythrophleum ivorense*	0	0	0	11.8	0	0
Lamiaceae	*Ocimum gratissimum*	0	11.2	0	0	0	0
	*Premna angolensis*	0	0	17.7	0	0	0
Musaceae	*Musa paradisiaca*	2.9	0	0	0	0	0
	*Musa sp.*	2.9	0	0	0	0	0
Piperaceae	*Piper umbellatum*	0	0	0	21.1	0	0
Poaceae	*Saccharum officinarum*	8.8	0	0	0	0	0
	*Cymbopogon nardus*	0	0	0	0	0	100
Rutaceae	*Citrus limon*	8.8	6.8	0	0	0	0
Siparunaceae	*Glossocalyx longicuspis*	7.5	0	0	0	0	0
Zingiberaceae	*Aframomum alboviolaceum*	0	0	0	32.9	0	0

The percent frequency of citation was calculated as the number of citations/total number of citations for all recorded species

Apart from Londji where no specific repellent plant was reported, the number of plant species used as repellent varied depending on locality. In fact, informants from Londji claimed to burn any plant to repel insect. This is a gap in ethnobotanical knowledge that needed to be spelled out. In Lolodorf and surroundings, 9 plants species were documented (*Elaeis guineensis*, *Chromolaena odorata*, *Canarium schweinfurthii, Cylicodiscus gabunensis*, *Glossocalyx longicuspis*, *Musa paradisiaca*, *Musa sp*., *Saccharum officinarum*, *Citrus limon*). The most used species were *Canarium schweinfurthii* (FC = 32.3%), followed by *Elaeis guineensis* (FC = 25.2%). *Musa paradisiaca*, *Musa sp.* and *Cylicodiscus gabunensis* (FC = 2.9%) were less mentioned as repellent. In Bipindi and surroundings, we recorded 6 species namely *Greenwayodendron suaveolens, Elaeis guineensis, Chromolaena odorata, Canarium schweinfurthii, Ocimum gratissimum*, and *Citrus limon*. Of note, *Canarium schweinfurthii* (FC = 36.9%) and *Chromolaena odorata* (FC = 17.9%) were the most cited species, compared to *Citrus limon* (FC = 6.8%). Three repellent species were reported in Kon-Yambetta, including *Canarium schweinfurthii* (FC = 49.8%), *Elaeis guineensis* (FC = 32.5%) and *Premna angolensis* (FC = 17.7%). In Dimako and surroundings four species were recorded including *Canarium schweinfurthii* (FC = 34.2%), *Afromomum alboviolaceum* (FC = 32.9%), *Piper umbellatum* (FC = 21.1%) and *Erythrophleum ivorense* (FC = 11.8%). Finally, in Babete, only *Cymbopogon nardus* was reported with FC value of 100% (Table 3).

### Plant parts used in the study sites and their modes of administration

**Fig. 2:**
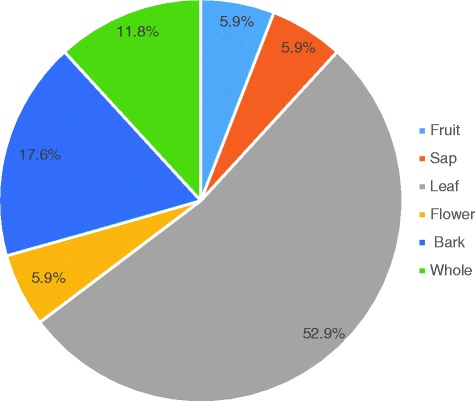
Plant parts used to repel insects. The information surveyed indicated the leaf as the preferred plant part used in repelling insects in the study areas

With respect to the parts of the plants used for repelling insects, results from the present study indicated that the local communities preferentially use the leaf (52.9%), followed by the bark, and whole plant (Fig. 2). This preference might be based on the fact that leaves are readily available or more likely that their volatile components have the desired activity. Viewing from another angle, this preference might also result from the desire of the communities to preserve the biodiversity on which they depend for food and remedies and from which harvesting plant barks or whole plants should rather contribute to the extinction.

From this study, it was recorded that communities adopt many modes of administration of plants products. Amongst those, 50% were reported to be burnt to produce smokes inside the houses, 31.2% mashed and thereafter applied on the body, and 18.8% hung in the houses. Of note, the only record from the Kribi-Londji site indicated the use of burning smokes from any plant to repel insects. Amongst the species reported, some were used in combination (Table 2). The flowers of *Elaeis guineensis* came out to be the primary ingredient to combine with the leaves of *Chromolaena odorata, Saccharum officinarum*, *Musa paradisiaca*, *Musa sp.*, and bark of *Cylicodiscus gabunensis* to afford blended repellent prescriptions.

Based on informants’ reports, the appropriate plant parts were collected only when needed and at any time. Moreover, no ritual was reported to be observed during plant collection. About the application time, the herbal preparations were preferably applied during sunset. The repellent effect was reported by the informants to be efficient mainly throughout the night. However, the specific dosage of plant materials used to repel insects was not clearly mentioned as they were administered as long as insects’ threat was pending.

## Discussion

This study attempted to record knowledge about plant species used as repellents against insects/mosquitoes in 6 localities of Cameroon to identify promising candidate plants that might be formulated as insect repellents. The results achieved indicated 16 plant species used by the people of Lolodorf, Bipindi, Dimako and their surroundings, and Londji, Kon-Yambetta and Babete of Cameroon to drive off insects, especially mosquitoes.

Most of the interviewed informants were men (86.8%) against 13.2% of women, similar to the pattern previously reported by Cheikhyoussef et al. [16], Bekalo et al. [17], and Okello and Segawa [18]. Besides, a meta-analysis on the continental level recently indicated significant differences in the knowledge of men and women in Africa when it comes to ethnobotanical knowledge. Additionally, women in rural Africa frequently collect plants for firewood purpose as one of the domestic activities they are responsible for [19].

The records showed that the local inhabitants (78.6%) had knowledge about the mosquito-repellent property of plants and their use. Most of the plants recorded (50%) were reported to be burnt to produce smokes inside the house, aligning with the claims of Pålsson and Jaenson [20] that indicated that burning plants might be effective in repelling insect. Similar results were equally obtained in Ethiopia and Tanzania, supporting this approach across communities as consistent to drive away mosquitoes [21, 22]. Biran et al, [23] equally reported mixed evidence especially for using firewood smoke to protect against mosquito bite. Despite the scarcity of data on how repellent smokes or their constituents act, the repellent activity of burned plants appears to be due to the release of specific volatile compounds, either already present in the fresh/dried plant or created during the combustion process [23, 24]. Such compounds like ß-ocimene have been previously showed to exert mosquito repellent activity [24]. Moreover, the smoke could act by disguising human kairomone cues targeted by insects and disrupting the convection currents essential for mosquito host location [22, 23].

Smashed plant samples for topical application (31.2%) and hung plants leaves inside the house (18.8%) were also recorded during our study as important modes of administration. These modes of repellent plants administration have been exploited for thousands of years by man, and are still in wide use throughout the developing countries [4]. Plants have been used for centuries in the form of crude fumigants where they are burnt to drive away mosquitoes and later on as formulations that are applied to the skin or clothes as firstly recorded in writings by ancient Greek [25], Roman [26], and Indian scholars [27].

Among the repellent plants species recorded in this survey, the main parts used were reported to be the leaf (52.9%) and the bark (17.6%). These findings are consistent with those of Zorloni et al. [28] who reported plants leaves and bark as predominantly used for tick control in West Ethiopia. The prominent application of leaves might be owing to the ready availability of their active constituents that are more likely volatile compounds. Indeed, plants that are commonly used for repellent properties are mostly those that contain essential oils, and when crushed or brushed against, leaves release strong odours of which some are pleasant, and some not so pleasant to insects.

The traditional use of plants or plant products against biting insects is a common practice in Africa [29]. Their insect-repellent effect observed may be attributed to their chemical composition [30]. Various active constituents with insects’ repellent activity have been previously reported from some of the checked plants species. Apart from *Glossocalyx longicuspis* and *Greenwayodendron suaveolens* for which very little has been reported as repellent properties, the other listed species or related species have previously been reported to display mosquito/insect repellent or insecticidal activities. Within this scope, the insecticidal activity of the bark extract of *Erythrophleum ivorense* was previously reported in the Ashanti region of Ghana [31]. Besides*, Erythrophleum ivorense* is resistant to fungi, dry wood borers and termites [32]. This denotes repellency/insecticidal properties that might be explained by the presence of pharmacologically active alkaloids in the bark and seed such as cassaine, cassaidine and erythrophleguine. However, it should be noted that high doses of the bark extract are extremely strong, rapid-acting cardiac poison in warm-blooded animals causing shortness of breath, seizures and cardiac arrest in a few minutes. Furthermore, the seeds are reported to be more toxic due to a strong haemolytic saponin which acts synergistically with the alkaloids [32]. Fresh bark of this plant was reported to be burnt by Mbamda (Bafia) people to repel in-house mosquitoes. Given the presence of toxic alkaloids in the bark, the resulting smokes are highly likely to be equally poisonous to insects and human, stressing the fact that it should be used with caution or simply discontinued.

*Canarium schweinfurthii* (Burseraceae), commonly known as African elemi or canarium, is a species of large tree native to tropical Africa*.* The African elemi tree is one of several sources of the economically useful oleoresin known elemi. In West Africa this resin is traditionally burned for fumigating dwellings and mixed with oil for body paint [33], and might likely exhibit insect repellent activity. *C. schweinfurthii* was the more cited by informants (FC > 32 when recorded) and was also previously reported to have insecticidal activity against *Callosobruchus maculates,* a major pest of cowpeas, green gram and lentils [34]. The authors assumed that this activity might be due to the presence of saponnins that have otherwise been found to affect the respiratory system of insect and to cause emetic effect by their detergent action. In addition, tannic acid found in this plant species was reported by David [35] to act as toxin and feeding deterrent to insects.

*Chromolaena odorata* (Compositae) is a tropical and subtropical species of flowering shrub in the sunflower family. It is native to North America, from Florida and Texas to Mexico and the Caribbean [36], and has been introduced to South America, tropical Asia, West Africa, and parts of Australia [37, 38]. It is sometimes grown as a medicinal and ornamental plant. It is used as a traditional medicine in Indonesia, Thailand, Malaysia and parts of Africa. In traditional medicine of Thailand, the plant is used for the treatment of wounds, rashes, diabetes, and as insect repellent. It has antifungal and antibacterial properties. It has previously been reported to have insecticidal properties against adult stage of *Periplaneta americana,* an omnivorous and opportunistic feeder which is the largest common species of pest cockroach especially in the tropics and subtropics [39]. Moreover, a survey undertaken in the Ashanti Region of Ghana revealed the use of *C. odorata* leaf as repellent with insecticidal properties [31]. *C. odorata* leaf and root have been shown to contain alkaloids, phenols, flavonoids, saponins, cardenolides, anthraquinones and tannins [40] that might elicit the insecticidal activity. Furthermore, this plant contains carcinogenic pyrrolizidine alkaloids that are toxic to cattle and can also cause allergic reactions [38, 41]. Previous findings also indicate that this class of alkaloids (retronecine type alkaloids) can elicit insecticidal activity [42]. This activity could also be linked to constituents such as flavonoids which are a class of phenolic compounds occurring naturally in this plant [43] and that have anti-feeding and attracting deterrent properties, thereby exerting toxic effects to insects, fungi, bacteria, nematodes and weeds [40].

*Citrus limon*, otherwise called lemon, is a small tree of the Rutaceae family that originated in Asia and is now grown commercially worldwide in tropical, semi-tropical, and warm temperate countries for the fruit, which is used fresh and in beverages and cooking, and is also used as a preservative due to its anti-oxidant properties. Lemon oil, obtained from the peel, is used as a wood cleaner and polish, and as a non-toxic pesticide. Traditional medicinal uses for the fruit, peels, and oil obtained from the seeds include treating fever and colic, and as an astringent and diuretic [44]. The topical repellent property of extracts from peels of *C. limon* against mosquitoes was previously reported by Effiom et al. [45]. In ancient medicine, Lemon citrus (*C. limon*) has long been used as natural insect repellent. Moreover, *C. limon* essential oil showed to be effective against mosquito larva, and to be also repellent against malaria vector, *Anopheles stephensi* in laboratory animal and human [46]. Kazembe and Chaibva [47] also showed that the whole extract of fruit peel and volatile oils had mosquito repellency against *Aedes aegypti*. On the specific compositional scale, citronellol, the most prominent component of *C. limon* essential oil, and linalool have been shown to be the main active ingredients of lemon in the distillate, and have also been identified among the main active ingredients of other botanical repellents such as citrosa and eucalyptus [46].

*Cymbopogon* (Poaceae), better known as lemongrass, is a genus of Asian, African, Australian, and Tropical Island plants in the grass family [48, 49]. Some species (particularly *C. citratus*) are commonly cultivated as culinary and medicinal herbs because of their scent, resembling that of lemons (*Citrus limon*). Common names include lemon grass, lemongrass, barbed wire grass, silky heads, citronella grass, fever grass, amongst many others. Research has shown that lemongrass oil has antifungal properties [50], but also the ability to repel some insects, such as mosquitoes. However, its oil is commonly used as a kind of trap to attract honey bees, working conveniently as honeybee's attractant pheromones (Beekeeping/Guide to Essential Oils). Essential oils from lemon grass principally contain geraniol and citronellol that are antiseptics, hence their use in household disinfectants and soaps. *Cymbopogon* spp. have a long history of use to repel mosquitoes and are as effective as the chemical insect repellent N,N-diethyl-meta- toluamide (DEET) [51]. *C. nardus* cultivation was introduced by Religious nuns at the Monastery Saint-Benoit in Mbouda-Cameroon and is used as mosquito repellent. Historically, it was used by the Indian Army to repel mosquitoes at the beginning of the 20^th^ century [52]. Essential oils and extracts from *C. nardus* and globally from the *Citronella* genus are commonly used as ingredients of plant-based mosquito repellents in commercial preparations [53]. Moreover, attempts to scientifically demonstrate the repellent efficacy of Citronella grass essential oils and formulations against mosquitoes and arthropods have been previously undertaken [53, 54]. Trongtokit et al. [55] also demonstrated that 100% essential oil of Citronella protects against *Aedes aegypti*, *Culex quinquefasciatus* and *Anopheles dirus* and related this activity to the presence of citronellol and related compounds.

*Saccharum officinarum* (Poaceae) or sugarcane, is a large, strong-growing species of grass in the genus *Saccharum*. It is cultivated in tropical and subtropical countries worldwide for the production of sugar and other products. *S. officinarum* has been shown to act as repellent, toxicant, and anti-feeding against a number of Coleoptera that attack stored food crops. The insecticidal property of *S. officinarum* bagasse-based lignin may be due to the presence of phenolic and alcoholic compounds [56]. Also, the efficacy of application of the stem juice of this plant on the skin as traditional personal protection method was evaluated against mosquito bites and general nuisance in Bolifamba, a rural setting of the Mount Cameroon area. The field evaluation of the skin application of the juice showed significant protection against mosquito bites though, less than the commonly used commercial diethyltoluamide [5].

*Ocimum gratissimum*, (Lamiaceae) commonly called clove basil, African basil is a native species of Africa, southern Asia, and the Bismarck Archipelago [57]. *O. gratissimum* is a common culinary herb in West Africa. Its essential oil contains eugenol and shows some evidence of antibacterial activity [58, 59]. This compound has otherwise showed anti-insect activity, therefore supporting the use of the plant as insect repellent. The authors alluded that this activity of eugenol was dependent on the structure of its phenolic hydroxyl [60]. The essential oil has potential for use as a food preservative [61]. An ethnobotanical investigation carried out in Bamenda, Cameroon reported *O. gratissimum* to be used as insect repellent [62]. This mosquito repellent plant is cultivated around houses for such purpose [63]. Also, people from many parts of Tanzania burn this plant to release smokes or hang it in houses to drive mosquitoes away [64]. Moreover, many other reports describe the insect repellent and insecticidal properties of *O. gratissimum* in field and laboratory trials, particularly against the main vectors of malaria and lymphatic filariasis [63, 65–67]. As asserted above, other studies also suggested that the insecticidal activities might be due to the presence of eugenol found in *O. gratissimum* [68, 69]. *Musa paradisiaca L.* and *Musa sapientum L.* (Musaceae) are mainly grown in the tropical and subtropical countries and are widely used for their nutritional values all over the world. The fruits as well as the other parts of the plant are used to treat different diseases in human in traditional medicines [70]. Our records indicated that *M. paradisiaca* and *Musa sp*. are used as insect repellents. This information was corroborated by previous reports showing that the dried leaf and stem of *M. paradisiaca* are burnt by the Ayta people of Porac, Pampanga province, Philippines, to drive off insects especially mosquitoes. In addition, laboratory investigations revealed the remarkable mosquitocidal activity of the petroleum ether root extract of this plant against *Ae. aegypti*, *An. stephensi* and *Cx. Quinquefasciatus* [71]. As well, 10% (w/v) concentration of *M. paradisiaca* leaf extract showed repellent protection of *Pterygota excelsa* wood against termite, *Odontotermes obesus* [72, 73]. Also, this extract exerted insecticidal activity against malaria vector *Anopheles stephensi* with 90% lethal effect after 24 h exposure [74]. Besides, the phytochemical studies of many parts of *M. paradisiaca* and *M. sapientum*, including leaf, fruit, peeled fruit, fruit pulp, fruit peel, flower, bracts, and scape previously revealed the presence of many chemical classes of components such as anthocyanins, Catecholamines, tryptophan, indole compounds, pectin, flavonoids and related compounds (Leucocyanidin, quercetin and its 3-Ogalactoside, 3-O-glucoside, and 3-O-rhamnosyl glucoside), tannins, Acyl steryl glycosides such as sitoindoside-I, sitoindoside-II, sitoindoside-III, sitoindoside-IV and steryl glycosides such as sitosterol gentiobioside, sitosterol myo-inosityl- β-D-glucoside, triterpenes such as cyclomusalenol, cyclomusalenone, 24- methylenecycloartanol, stigmast-7-methylenecycloartanol, stigmast -7-en-3-ol, lanosterol and β -amyrin [70, 75]. Among all these chemical classes, there are some that might elicit biological activities sustaining the repellent feature of *Musa* sp. For example, compounds such as anthocyanins have been suspected to act in a vast array of plant/animal interactions, including attraction of pollinators and frugivores, as well as the repellence of herbivores and parasites [76]. Also, saponins that derive from a sugar moiety glycosidically linked to a hydrophobic aglycone which may be a triterpene or a steroid have been shown to trigger plant resistance against insects [77]. The mechanisms underlying the action of these compounds against insects are based on their various biological properties. They have membrane-permeabilising, haemolytic, antioxidant, anti-inflammatory, immunostimulant and anticarcinogenic activities, and can affect feed intake, growth and reproduction in animals, and can be used as fungicides, molluscicides and pesticides, as well as against some bacteria and viruses. They can also increase mortality levels, lower food intake, weight reduction, retardation and disturbances in development, and decrease reproduction in insects [78].

The main hypotheses are therefore that saponins could either make the food less attractive to eat (repellent/deterrent activity), bear digestive problems, causing moulting defects or having toxic effects on insects. Another class of compounds such as flavonoids are small molecular secondary metabolites synthesized by plants with various biological activities. Due to their physical and biochemical properties, they are capable of participating in interactions with other organisms. Both flavonoids and isoflavonoids protect plants against insect pests by influencing their behavior, growth and development [79, 80]. In this line, Naringenin, hesperetin-7-O-rutinoside and quercetin-3-O-rutinoside were reported to stimulate oviposition in swallowtail butterfly Papilio on young leaves of citrus plants [81]. Also, Chrysin, Kampferol, and 3,7- dimethylether quercetin were found to exert remarkable repellent action against house flies (*Musca domestica*) [82]. Another interesting example is the tannins that were assessed as repellents in other studies. Tannins have a strong deleterious effect on phytophagous insects and affect the insect growth and development by binding to the proteins, reduce nutrient absorption efficiency, and cause midgut lesions [83].

*Elaeis guineensis* (Arecaceae) commonly called African oil palm or macaw-fat is the principal source of palm oil. It is native to west and southwest Africa, specifically the area between Angola and the Gambia. An innovative phytodrug (API-PALU) was recently formulated from the crude alkaloids extract of this plant and is extensively used in West Africa to treat malaria (http://www.africanews.com/2016/06/24/beninese-wins-100000-for-innovation-of-anti-malaria-drug/). The innovator recently won many prizes including the 1^st^ IPA 2016 prize (http://www.africanews.com/2016/06/24/beninese-wins-100000-for-innovation-of-anti-malaria-drug/). *E. guineensis* was one of the most cited species recorded in our survey and has been reported in Africa as reducing mosquito biting activity when used as repellent [84]. In fact, smokes from burned infructescences of *E. guineensis* were reported to reduce the numbers of mosquitoes indoors at night. Also, a field experiment using these smokes showed 69% repellent activity [20]. As well, *E. guineensis* palm nut (lotions and creams) and oil were shown to reduce significantly the number of bites by *Simulium damnosum* and *Anopheles gambiae* [85, 86]. Moreover, flowers of *E. guineensis* were recorded in this study to be the primary ingredient to combine with the leaves of *Chromolaena odorata, Saccharum officinarum, Musa paradisiaca, Musa sp.,* and the bark of *Cylicodiscus gabunensis* to repel insects and mosquitoes. The phytochemical composition of the leaf and flower of *E. guineensis* could justify the repellent activity elicited by this plant. Indeed, a recent study recently revealed the presence of phenolic compounds, flavonoids, tannins, coumarins, alkaloids, saponins, terpenoids and steroids, and carbohydrates in the leaf of *E. guineensis* [87]. Another previous study identified p-methoxyallylbenzene (estragole) as the predominant (~95%) constituent of essential oils from both male and female flowers of *E. guineensis* [88]. Many of the secondary metabolites classes identified in the leaf have already been reported as having deleterious effect on insects [40, 42, 43, 56, 77, 79–81]. From recent studies, it was demonstrated that the main component of *E. guineensis* flowers essential oils (estragole) has strong repellent effect to three of the major grain pest insect species, *viz. Rhyzopertha dominica, Sitophilus zeamais,* and *Tribolium confusum.* Interestingly, estragole and (E)-anethole (a related phenolic compound also found in essential oils) showed a strong synergistic co-repellent effect against *R. dominica* [89]. These latter findings further emphasize the potential of components of single and/or mixed plants to act synergistically to repel insects.

*Cylicodiscus gabunensis* (Leguminosae) is a large tree, common in the rainforests of West and Central Africa. The stem bark is used to treat jaundice and malaria among other diseases. It contains triterpene saponins, cylicodiscus acid, a dihydroxy-pentacyclic triterpene carboxylic acid and cyclodione, a dimeric diterpene [90]. There are however, no references in the literature describing the repellent application of the stem bark of this plant despite its strong odour [91]. Nevertheless, *C. gabunensis* wood is very resistant to worm and insect attack [92]. However, the strong odour exhaled by *C. gabunensis* stem bark is likely elicited by its terpenoids content, and might otherwise justify its repellent effect against insects.

Our study also reported *Piper umbellatum* (Piperaceae) to be used as mosquitoes/insects repellent. It is commonly known as English cow-foot leaf (Sierra Leone), *Fula-Pulaar* (Guinea); Poponidagui *(*Sierra Leone). It is an upright shrub to 2 m high in moist shady places occurring from Guinea to Cameroon, and widespread throughout the tropics where it is used as condiment, and also considered a fetish as well as a medicine to treat a vast array of ailments including but not limited to pain, arthritis, rheumatism, fever, diarrhoea, dysentery, venereal diseases, hemorrhoids [93].

Previous studies have reported the repellent and insecticidal activities of the essential oil of *P. umbellatum* when investigated against grain storage pest insects, bean weevil (*Callosbruchus maculatus*) and rice weevil (*Sitophilus oryzae*), suggesting its suitability for insect pest control [31, 94]. As well, when *P. umbellatum* fresh leaves are crushed and rubbed on the skin there is an effective though transient (1-2 h) repellent effect, particularly against mosquitos. The active agent was found to be the essential oil consisting of aldehydes, ketones, and phenols in addition to the principal constituents, *viz.* cadinene, caryophyllene, and phallandrene. The repellent effect may be prolonged by mixing the crushed leaves with oils or glycerin-alcohol [94, 95]. On another hand, extracts from closely related species *Piper nigrum*, *Piper guineense*, and *Piper tuberculatum* contain isobutyl amides, and other plant secondary metabolites that act as neurotoxins and showed repellent activity against insects [96].

*Premna angolensis* (Lamiaceae) has also been reported to have repellency potential [97]. The various *Premna* species are well known for their medicinal properties and their further phytochemical investigations resulted in the isolation of secondary metabolites including iridoids and their glycosides, diterpenoids, sesquiterpenoids, triterpenoids, flavonoids, isoflavones, lignans, xanthones and other classes of compounds [97] that might be involved in the repellency properties. Many plants from Lamiaceae family have been found to be effective against a variety of mosquito vectors. For instance, the crude aqueous, chloroform and methanol extracts and essential oils from the leaves of *P. latifolia* showed mosquito larvicidal efficacy against the fourth instar larvae of *Aedes albopictus* Skuse (Diptera: Culicidae) [98]. *P. angolensis* and *P. quadrifolia* leaves are burned and used as fumigant in the attics of cereals against pests. Besides, their essential oils displayed insecticidal and repellent effects against *Sitotroga cerealella*, an insect pest of rice stocks [99].

## Conclusion

This study that aimed at documenting insects repellent plant species used by indigenous populations of 6 localities of East, South, West and Centre regions of Cameroon has successfully identified indigenous knowledge on insects’ repellent plants. There was consistency in documented information with previous reports for most of the plants cited, and the results achieved have potential as baseline for further scientific investigation to strengthen the concept of plant-based mosquito repellents. These findings will also provide validation to the ethnobotanical knowledge of the targeted communities. Besides, it was recorded that the bark of *Erythrophleum ivorense* that contains toxic alkaloids is burnt to repel mosquitoes in houses. Given its toxicity, this plant should be used with caution or simply discontinued. Further laboratory investigations are ongoing and will contribute to establish the cidal or static effects of extracts from selected plants against mosquitoes in particular and insect pests in general.

## Additional file

Additional file 1: Ethnobotanical survey of insect/mosquito repellent plants. Interview respondents were identified and further questioned face-to-face using a semi-structured questionnaire. Responses to all questions were recorded following a sequential guideline. (DOC 53 kb)

## Abbreviations

FC: Frequency of citation
