# Supplementary material for: Ethnobotanical survey of medicinal plants used as insects repellents in six malaria endemic localities of Cameroon
Source: J Ethnobiol Ethnomed. 2017 Jun 8;13:33. doi: 10.1186/s13002-017-0155-x (PMC5465592; doi:10.1186/s13002-017-0155-x)
Supplement: Additional file 1: — Ethnobotanical survey of insect/mosquito repellent plants. Interview respondents were identified and further questioned face-to-face using a semi-structured questionnaire. Responses to all questions were recorded following a sequential guideline. (DOC 53 kb) [file 13002_2017_155_MOESM1_ESM.doc]

**FICHE D’ENQUETE/ *SURVEY FORM***

**THEME**:

**Enquête ethnobotanique sur les plantes à effet répulsif des moustiques/insectes**

***Ethnobotanical survey of insect/mosquito repellent plants.***

1. INFORMATIONS LEGALES/ *LEGAL INFORMATION*

•Localité /*Locality*:…………………………………………………………………………………

•Communauté (ethnie)/ Quartier/ village (*Community/Town/village*) :……..………..………………………………………………………

•Nom de l’informateur (*Informant’s name*): …………………………..................Age:…………

•Collecteur (*Investigator*):…………………………………………………………………………..

•Interview N°………………………………………….

•Date de récolte (*Date of collection*):……………………………………………………………….

•Type de végétation (forêt, savane, etc)/ *Habitat* (*forest, savanah, etc*): …………………………

B. QUESTIONNAIRE

1. Connaissez-vous des plantes utilisées pour chasser les moustiques/insectes/ *Do you know plants that are used to repel mosquitoes/ insect* ?..……… ………………………………………

2. En avez-vous qui poussent (ou qui sont cultivées) dans votre village/ *Are some of these plants growing (grown) in your village ?...................................................................................................*

3. Quelles sont ces plantes que vous utilisez contre les insectes/moustiques ? /*What are these plants you use to repel insects/mosquitos ?*

a. Type de plante/ *Type of plant* (Tick to select)

O Arbre/*Tree;* O Arbuste/*Shrub;* O Herbacee/*Herb;* O Liane/*Liana;* O Plante aquatique/*Aquatic plant;* O Autres/*Other* : ……………………………………………………..

…..………………………………………………………………………………………………..…

b. Nom(s) vernaculaire(s)/*Local name(s)* (*specify dialect/language*):

………………………………………………………………………………………………………………………………………………………………………….......................................................

c. Nom(s) commun(s)/*Common name(s)*:………………………………………………………….

………………………………………………………………………………………………………

d. Nom scientifique/ *Scientific name* (genre/*genus*, espece/*species*, famille/*family*):

………………………………………………………………………………………………………

Synonyme(s)/*Synonym(s):*…………………………………………………………………………

e. Partie utilisée/*Plant part used* (specify whether fresh or dried):………………………..

O Ecorce de tronc/*Stem bark;* O Tige/*Stem;* O Feuille/*Leaf;* O Racine/*Root;* O Ecorce de racine/*Root bark;* O Fleur/*Flower;* O Fruit; O Graine/*Seed;* O Plante entière/*Whole plant* small; O Autres/ *Others:……………………* ………………………………………............

4. Dans quelle formulation utilisez-vous la plante contre les insectes/ moustiques ?/*How do you prepare* *plants to be used against insects/mosquitos ?*

………………………………………………………………………………………………………………………………………………………………………………………………………………………………………………………………………………………………………………………………………………………………………………………………………………………………

5. Quel est le mode d’administration de la plante contre les insectes/ moustiques?/ *How do you administer the plant against insects/mosquitos ?...............................................................................*

*…………………………………………………………………………………………………………………………………………………………………………………………………………………………………………*

6. Comment cette plante agit-elle sur les insects/moustiques ? /*How does the plant affect insects/mosquitoes ?*

O Elle les tue/*Plant kills them;* O Elle les chasse/*Plant repels them;* O Elle les endort/*Plant bores them;* O Autres/*Others* ……………………………………………………………………

7. Utilisez-vous la plante en association avec d’autres ingredients ? /*Do you use the plant in combination with other ingredients?..................................................................................................*

Si oui, lesquels?/*If yes, list them…………………………………………………………………................*

*……………………………………………………………………………………………………………………*

S’il s’agit de plantes, décrivez-les/*If the ingredients are plants, describe them:*

Nom (genre, espèce, synonyme, famille)/ *Name (genus, species, synonym, family)*: ………………………………………………………………………………………………………

Nom vernaculaire (dialecte)/ *Vernacular name (dialect)*:………..…………………………………

Partie utilisée/ *Part used*:…………………………………………………………………………....

8. Organes collecté pour les essais au laboratoire/*Plant parts collected for laboratory screening*

………………………………………………………………………………………………………

9. Autres informations utiles/*Other useful notes*

………………………………………………………………………………………………………………………………………………………………………………………………………………………………………………………………………………………………………………………………………………………………………………………………………………………………
